# Induction of Resistance Against Isotransplantation of Virus-Induced Myeloid Leukaemias

**DOI:** 10.1038/bjc.1963.71

**Published:** 1963-09

**Authors:** G. Pasternak, A. Graffi


					
532

INDUCTION OF RESISTANCE AGAINST ISOTRANSPLANTATION

OF VIRUS-INDUCED MYELOID LEUKAEMIAS

G. PASTERNAK AND A. GRAFFI

From the German Academy of Science8, Institute for Experimental Ca?icer Research,

Berlin-Buch, Germany

Received for publication April 8, 1963

ALL the experiments carried out in various laboratories since the publicatioils
of Foley (1953) and Prehn and Main (1957) concerning the antigenicity of experi-
mental tumours confirmed that sarcomas induced by carcinogenic hydrocarbons

contain specific antigens (Re've'sz, 1960; Klein et al., 1960; Koldovsk', 1961a;

y

Old et al., 1961 ; Pasternak, Horn and Graffi, 1962a). This conclusion, for example,
resulted from experiments in which resistance against cell inocula of the same
isologous tumour could be produced by pretreatment of mice with X-ray irradiated
tissue suspensions of a tumour or after ligation and following regression of a sub-
cutaneous or an intradermal tumour graft.

Since immunologic cross-reactions, even between tumours which were induced
by the same carcinogen, could not be observed by most of the investigators (Klein
et al., 1960; Prehn, 1961 ; Feldman, Globerson and Yaffee, 1962; Pasternak et al.,
1962b, in contrast to Koldovsk', 1961b) for each of these tumours, individually
specific antigens may be suggested.

A specific antigenicity could also be demonstrated for the tumours in mice
induced by the polyoma virus. In contrast to the carcinogen-induced tumours at
least a great number of those tumours in mice induced by this virus, seem to con-
tain the same cellular antigen (universal antigen) (Habel, 1961 ; Sj6gren, 1961),
since resistance against isologous tumour grafts developed both after treatment
of the animals with the virus and after homotransplantation of the polyoma
tumours. Virus-neutralizing antibodies did not affect the growth of the tumours.

These differences concerning the antigenic properties of tumours of different
aetiology suggested an investigation of the antigenicity of virus-induced leukaemias.

The results of earlier experiments (Pasternak et al., 1962c) already indicated the
possibility of an active immunization at least against one of the two transplantable
myeloid leukaemias of the strains C57BI and CBA tested. Furthermore, Klein,
Sj6gren and Klein (1962) were able to prove in extensive experiments that also in
mice a state of relative resistance may be produced against lymphomas, induced
by the Gross agent, if the recipients were pretreated with homografts of these
leukaemias or with sub-threshold cell doses of the same isologous lymphoma. The
partial or complete cross-reactions obtained for the different lymphomas, indicate
that similar or identical antigens may be found in these virus tumours which are
however different from those of polyoma tumours.

In the present paper the existence of specific antigens in isologous grafts of the
virus-induced myeloid leukaemia of mice (Graffi et al., 1954) will be described.

533

INDUCTION OF RESISTANCE AGAINST LEUKAEMIAS

Since in previous experiments (Pasternak and Graffi, 1961) common antigenic
components in viruses of the myeloid and the lymphatic leukaemia could be
proved, we were especially interested in the antigenic relations between the
different types of leukaemias induced by these viruses.

MATERIALS AND METHODS

Experimental animals and tumour donors were inbred mice of the strains CBA,
C57BI, XVII and AKR. In one of the experiments Fl-hybrids produced by crossing
XVII females and CBA males were used. AR animals were taken from a breed
which is maintained by brother-to-sister mating (Pasternak and Gryschek, 1962).
The skin graftings performed showed the homogenicity of the strains used; the
survival of the grafts exceeded 150 days.

The leukaemias used for the experiments and their data are presented in Table
1. All of the myeloid leukaemias were induced by injection of cell-free filtrates of

TABLE I.-Some Data on the Leukaemias used for the Experiments

Number of

transfer

Latency               generations
period    Strain of  in isologous

Designation     Induced by      (days)     origin       hosts       Histological type

PL 1462      Virus of mye-       161       CBA          46        Myeloid leukaemia

loid leukaemia

L 129/6d                         158      C57BI         17

(Chloroleukaemia)

L 112b                          206       C57BI         21        Myeloid leukaemia
L 184/3a                         119      xvii          14

L 1205         Gross agent      303       AKR            2           Lyniphatic

(spontaneouslv                                          leukaemia

developed)

L 1061            31 ?           326      AKR

L 1937            311,           216      AKR

virus-induced leukaemias in newborn mice. These leukaemias were transplanted
to the original strain. In these experiments each of the leukaemias was tested in
different generations of transplanting, in order to determine possible changes in
the antigenic composition. The lymphatic leukaemias developed spontaneously
in mice of the AKR strain containing the Gross agent. Lymph nodes and spleens
from animals with leukaemias L 1061 and L 1937 were transplanted for immuniza-
tion purposes exclusively to homologous recipients; in addition the leukaemia
L 1205 was further transplanted to isologous recipients.

In each of the transplantation or immunization experiments performed with
leukaemic materials the sex of the animals was taken into consideration to exclude
reactions against Y-linked H-antigens. Leukaemias however which originated
from females were also transplanted to males. All experimental animals were of
uniform weight ; their age ranging from 3 to 4 months. For purposes of immuniza-
tion the animals were treated as follows :

(1) At intervals of two weeks isologous recipients received 2 to 3 subcutaneous
injections of a suspension from leukaemic tissue (0-2 to 0-3 ml. each), which had
been irradiated in vitro at a dose of 14500 r. For that purpose the tissue (intra-
muscular graft, lymph nodes or spleens) was cut into small pieces with scissors and

534

G. PASTERNAK AND A. GRAFFI

slightly homogenized by hand in a small quantity of a phosphate-buffered isotoiiic
saline solution using a Potter glass homogenizer. Previous to irradiation the sus-
pensioii which contained for the most part intact cells aiid cell aggregates was
further diluted with a saline solution to a ratio of I : 4 as compared to the initial
weight of the material.

(2) Under similar experimental conditions homologous recipients received sub-
cutaneous injections of the same but non-irradiated tissue suspension in doses of
0.2 to 0-3 ml. In general one single treatment was sufficient to produce resistailce
against isologous grafts of myeloid leukaemia.

(3) Isologous recipients were treated by subcutaneous injections of sub-

threshold cell doses (in some cases up to 5 X 104 cells). Only those animals were

taken for the tests which did not show any subcutaneous tumours or in which the
tumours regressed after temporary growth.

Test injections for the determination of the induced resistance were made either
1-7 days after treatment with the irradiated tissue suspensions or at particular
intervals after the animals had been pretreated with sub-threshold cell doses. The
cell suspensions were obtained by trypsination (0- 15 per cent solution) of the tissue
material (Graffi, Pasternak and Horn, 1962). After vital staining with trypan blue
the cells were counted and adjusted to the doses required.

The animals were pretreated by left lateral subcutaneous injectioils while the
test injections were given subcutaneously on the right side. In some experimeilts
the intravenous mode of administration was chosen. The results of experiments
presented in the different tables include the number of resistant animals in com-
parison with the total number of test animals which were fit for evaluation.

RESULTS

Induction of resi,3tance against graft8 of viru8-induced inyeloid leukaemia-8 by pi-e-

treatment of mice with isoloyou8 tumour material

The results summarized in Table 11 indicate that 78-9 per cent of the pre-
treated animals are resistant to cell inocula of isologous leukaemias as compared
with 12-0 per cent of the untreated controls. This difference is statistically highly
significant and implies that the pretreatment gave rise to an active defence of the
host. The effect was marked in all three strains of mice, on each of which one type
of myeloid leukaemia was tested. It should be mentioned, however, that the
method emploved for immunization plays an essential role for producing this
resistance. After treatment of CBA niice with irradiated tissue of leukaemia PL
1462 it was at no time possible to demonstrate a resistance against grafts of this
isologous tumour, while mice of the strain XVII revealed a strong resistance having
received the same treatment with tissue material of leukaemia L 184/3a-both
after subcutaneous and intravenous test injections. That the leukaemia PL 1462
contains specific antigens and that CBA mice were able to react against this type
of leukaemia results from the fact that after pretreating the animals with sub-
threshold doses of cells, the number of takes in the test group was noticeably
diminished in comparison with those in the control group.

The results obtained after immunization of C57BI mice with tissue material
from the leukaemia L 129/6d indicate that an active resistance was produced by
using both methods of immunization. Detailed data on the varying effectiveness
of certain methods of immunization will be published later.

INIDUCTION OF RESISTANCE AGAINST LEUKAEMIAS

01', 3 5

--4
0

O

0

Ile             c' ;. "t 't    " 0      O 0

b]D

-d4 C>

v
00          aq    (m C>    t-

t- 00   to O               00 00

eQ                                                              04

4-D

ce

f4-               r-.4

4") > >

.4a (=>

e4 EN                         C        w O      C 0
t2                            -410     c'

zt               O X X           O

O     C)           X          x      x x

0 0   XO xo    10          xo     xo xo

0

eQ                                               Ca

Z3

14?

4Q?

o

0                                     0
4Z
0 >

0        0 >

-'-q       4.

ce

0                 0
0

-4        -lb, '.4 - -4

4a I                  (S)
C)       r. "coo

(D 0

t-4
O's

eQ.                                                                        0

4Q.

0 O.bp

0

O

Cs

C4-4   ;-4                               CS
0     0

4a

co

4---, P.,                                                4a
0

0

xo
0
0

536

G. PASTERNAK AND A. GRAFFI

Induction of resistance against isologous grafts of virus-induced myeloid leukaemias

by pretreatment of mice, with homologous grafts of myeloid leukaemias (im-
munologic cross-experiments)

Inbred mice, in which homologous grafts of virus-induced myeloid leukaemias
have regressed, reveal a relative resistance to isologous grafts from leukaemias of
the same origin. This conclusion can be drawn from the results of experiments
presented in Table 111. In addition the table contains the results of cross-experi-
ments carried out with leukaemias L 129/6d and L 112b on C57BI mice. C57BI
mice in which resistance was produced against cell inocula of the leukaemia L 129/
6d were also resistant to isologous grafts of the leukaemia L 112b. The final results
of 63-7 per cent of resistant animals in the test group as compared with 33-3 per
cent in the control group is statistically highly significant.

Experiment-3 to produce resistance against isologous grafts of virus-induced myeloid

leukaemia8 by pretreatment of mice with homologous grafts of virus-conditioned
lymphatic leukaemias

The three experiments hitherto performed utilized different lines of the lym-
phatic leukaemia for the pretreatment of mice of the strains XVII and C57BI.
They showed the progressive growth of isologous grafts of myeloid leukaemias on
these animals. In comparison with the controls even a certain growth-stimulating
effect was found in mice subjected to treatment. While homologous grafts of
myeloid leukaemias induce an active resistance against cell inocula of isologous
leukaemias having the same origin, homologous grafts of spontaneous virus-induced
leukaemias of the AKR strain are not able to produce resistance against grafts of
myeloid leukaemias. The percentage of resistant animals in the test group was
1-9 as against 7-1 in the control group.

TABLIF, IV.-Results of the Experiments to Produce Resistance Against Isologous

Grafts of Virus-induced Myeloid Leukaemia8 by Pretreatment of Mice with
Homologous Grafts of Virus-conditioned Lymphatic Leukaemias

Line of

leukaemia  (Genotype  Designation of  Number

Mouse strain    used for     of        challenge      of cells  Immunized   Control

inoculated   pretreatment  origin)    leukaemia    inoculated    group      group

xvii          L 1205    (AKR)        L 184/3a       5 X 104    0/19*      0/10*
C57BL         L 1061    (AKR)        L 129/6d         105      1/20       2/9
C57BL         L 1937    (AKR)        L 129/6d       3 x 10,5   0/13       0/9

Total : 1/52       2/28
Pereentage of resistant animals: 1,9    7,1

z2          1,5
p         >0,3
Number of resistant animals over the total number inoculated.

DISCUSSION

The experiments performed indicate the possibility of producing resistance
against isologous grafts of virus-induced myeloid leukaemias by pretreatment of
mice with cells or tissues of transplantable leukaemias of the same origin. Such a
resistance is ascertainable if about 104 to 105 cells are given subcutaneously or
intravenously; the percentage of resistant animals in the test group is significantly

INDUCTION OF RESISTANCE AGAINST LEUKAEMIAS

537

"e

zt

?b7

q*n

Iz

Co
I

CO e-,
w00

w   I?Q

pj;:t

4Z

pq

--4

0 ?=, -?--

0 o c) o=0M Ot- co- to

o -I -4 aq M'" -4 -4 r--q "4 aq -4 P-4 4m
t., -- -- -- -- --- -- -- -- ---
to = =m =cq c? - -04 to 0 (= 0mm

-4                10m

. . t- -1

m O
M -4

v
C)

-4   t'!
'.4  =
eq =
P-4

..   .. CQ

.--i  m    N     04

0 44
-4-D  es
0

E--l

0
C3

-4-?
9
m
m
(1)

C4-4
0

ea
-4-)
0
Q
(L)
0 .

(1)
P.,

19

.- P- o m     oo =   c> o m   o =   0=

o 0 -4 _q -4 -4 cq 1-4 1-4 cli -14 m aq --I
0 0 -- -- -- -- -- , -- -- --

r-4 O = cq = 10 m t- P-4 - Cl = 10
bo -4 -4 -4 -4  q        _4 -4

r.
0

4.4 -4
o I
0 1-1

O.a
0

P4 5

10

Cs

. . . . . . . . . . . .

,:?    :?  (?     ?    C?    (?   C?             (?      C?   (?

M M        M -4 M M M                 M .-        M cli M

10

ui C)

c -4

,--q X

m

" " . . . "

o o o c) 0 0 "

'.4 q -4 -4 '.4 --4 0
x x x x x x -

10 xo IfD in 10 10

0 0 10

I C) o

X -4 "

aq

r-4

Go
0

.kQ --4

(D  F-4

P.C4.    M

0 .4.?

?4 P4

C4-4

0        C3

(D .,q
m ?
.2 0 (D

-4'?,    as

Ca --, 14

O Ca :z

m 14 (3)
m (t) --I
(1)
1::?

T$

(M
N
P-4

?-q

aq
to

F--4

?4

PLI

0        1.01

4..4

0

4-1

C3    -d 1?   cq cq Ca   Ca T$ cq aq Tl
m 4 co w = = VD = w w = CD

cq     =   "ni .t ldq ,.d4 (M  "I vt =

,it           P-4 -4            -4 r--q

00 -4 cq cq         00   00 aq        aq

-4 "-.q -4 -4 ?44    --I P" -44    4  r-4

4  ?.q  4  4  P? P-4 4   ?-4 4  41 4, g

PLI

P?
u
x
?-q

1-4
pq
t-
xo
U

1-4
pq
t-
xo
C-)

4-4

O.-

538                     G. PASTERNAK AND A. GRAFFI

higher than in the group of the controls which had not beeii immunized. The
treatment of coiitrol animals with normal tissues was considered dispensable since
previous experiments (Pasternak et al., 1962a) had proved that the growth of
isologous tumour grafts was not influenced by this pretreatment.

Considering these results obtained with myeloid leukameias aiid the findings of
Kfein et al. (1962) resulting from work with lymphatic leukaemias and takilig into
coiisideration studies by Sachs (1962), it may be assumed that in geiieral virus-
induced leukaemias in mice reveal a specific antigenicity.

In the experiments of Sachs, who tested leukaemias induced by the Molonev
virus, a resistance against isologous grafts of these leukaemias was also produced
by pretreatment of mice with a virus-containing tissue culture fluid. This would
be in approximate accordance with the effect observed by Habel (1961) and Sj6gren
(1961) for the polyoma virus which showed that the growth of polyoma tumour
grafts is inhibited if adult mice had been pretreated with this virus. Klein et W.
(1962) were not able to detect cross-reactions between the leukaemias and the
polyoma tumours, a fact indicating the existence of different antigens. Yet, the
mode of induction of a new cellular antigen by the action of either a DNA-virus
or a RNA-virus seems to be most similar. The present results do not allow assump-
tions as to whether the specific antigenicity is confined exclusively to the malignant
cell. And, similarly, the role which viral antigens play in producing a resistance
to isologous grafts of leukaemias is not thoroughly clear. In a previous paper
(Pasternak and Graffi, 1961) we were able to show that the virus of myeloid
leukaemia is inactivated both by antisera against cell-free filtrates of myeloid
leukaemias and by similar antisera against lymphatic leukaemias (induced by the
Gross agent). These viruses seem to contain common antigens though the cellular
antigens of both types of leukaemia are probably different as can be seen from the
absence of cross-reactions described for the present experiments. It remains to be
seen which antigenic correlations will be found to exist between other types of
leukaemias induced by different viruses and those already tested.

The absence of cross-reactions between virus-induced lymphatic leukaemias
and leukaemias of different aetiology (Slettenmark and Klein, 1962) is a proof of
the exceptional position virus-induced leukaemias occupy. Judging from the results
hitherto available the existence of a universal antigen of leukaemias appears to be
doubtful.

SUMMARY

Resistance against isotransplaiitation of virus-induced myeloid leukaemias of
mice was produced by pretreatment of the animals with irradiated tissue suspen-
sions of these leukaemias or by inocWation of small doses of viable cells prepared
from isologous leukaemic material or by homotransplantation of leukaemic tissue
of the same origin. Mice treated under equal experimental conditions with homo-
logous grafts of spontaneous lymphatic leukaemias of the AKR strain did not
develop any resistance against isotransplantation of myeloid leukaemias. The
conclusion drawn is that the cells of the two types of leukaemias contain different
antigens.

REFERENCES

FELDMAN, M., GLOBERSON, A. AND YAFFEE, D.-(1962) Abstracts of the 8th International

Cancer Congress, Moscow, p. 15.

FOLEY, E. J.-(1953) Cancer Res., 13, 835.

INDUCTION OF RESISTANCE AGAINST LEUKAEMIAS               539

GRAFFI, A., BIELKA, H., FEY, F., SCEURSACH, F. AND WEISS, R.-(1954) Naturwi88-

en8chaften, 41, 503.

Idem, PASTERNAK, G. AND HORN, K.-H.-(1962) Acta biol. med. germ., 9, 318.
HABEL, K.-(1961) Proc. Soc. exp. Biol., N.Y., 106, 722.

KLEIN? G., SJ6GREN, H. 0. AND KirEIN, E.-(1962) Cancer Re8., 22, 955.

IdeM, SJ6GREN, H. O., KLEIN, E. AND HELLSTR6m, K. E.-(1960).Ibid., 20,1561.
KOLDOVSKY, P.-(1961a) Folia biol., Praha., 7, 115.-(1961b) Ibid., 7, 162.

OLD, L. J., BENACERRAF, B., CLARKE, D. A., CARswELL, E. A. AND STOCKERT, E.-(1961)

Cancer Re8., 21, 1281.

PASTERNAK, G. AND GRAFFI, A.-(1061) Z. Naturfor8ch., i6b, 73.
Idem AND GRYsCHEK, G.-(1962) Z. Ver8wh8tierkunde, 1, 184.

Idem, HORN, K.-H. AND GRAFFi, A.-(1962a) Acta biol. med. germ., 9, 302.-(1962b)

Ibid., 9, 306.-(1962c) Ibid., 9, 314.

PIELIEHN, R. T.-(1961) J. nat. Cancer Imt., 26, 223.
Idem AND MAm, J. M.-(1957) Ibid., 18, 769.
RE'vE'sz, L.-(1960) Cancer Re8., 20, 443.

SACHSI L.-(1962) J. nat. Cancer.1n8t., 29, 759.
SJ6GREN, H. O.-(1961) Virology, 15, 214.

SLETTENMARK, B. AND KLEIN, E.-(1962) Cancer Re8., 22, 947.

				


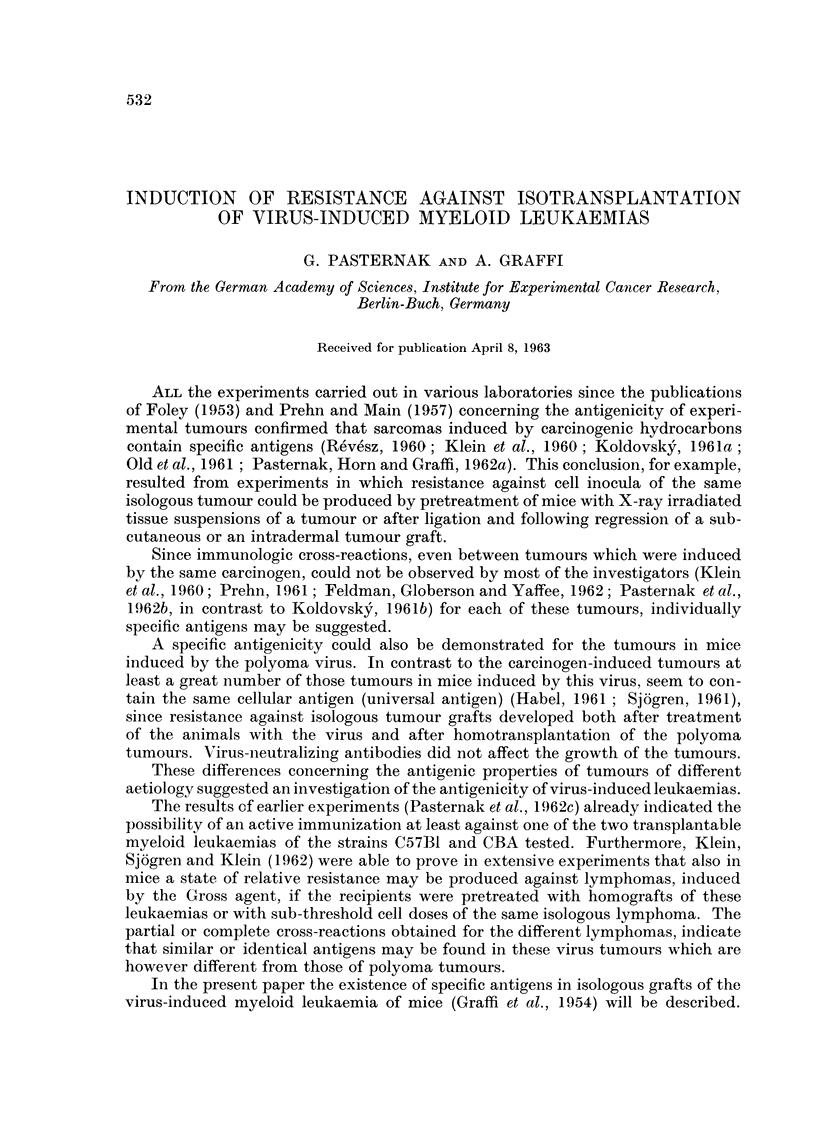

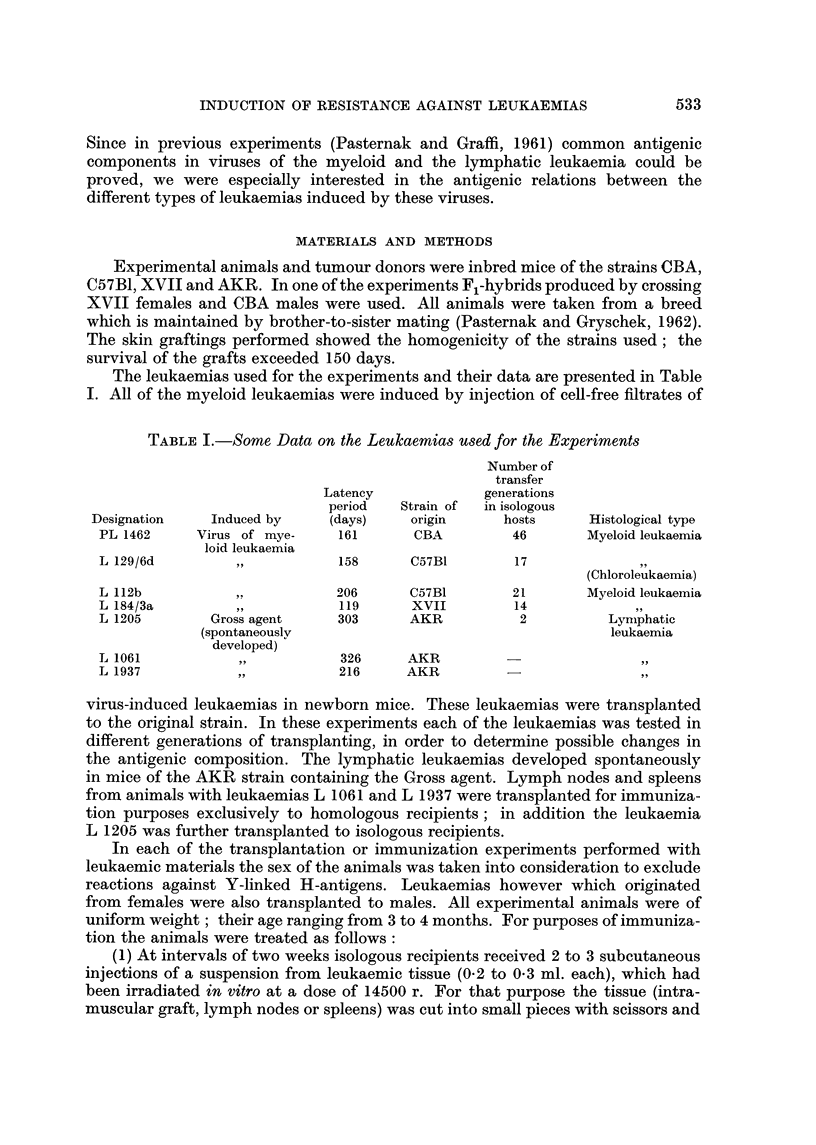

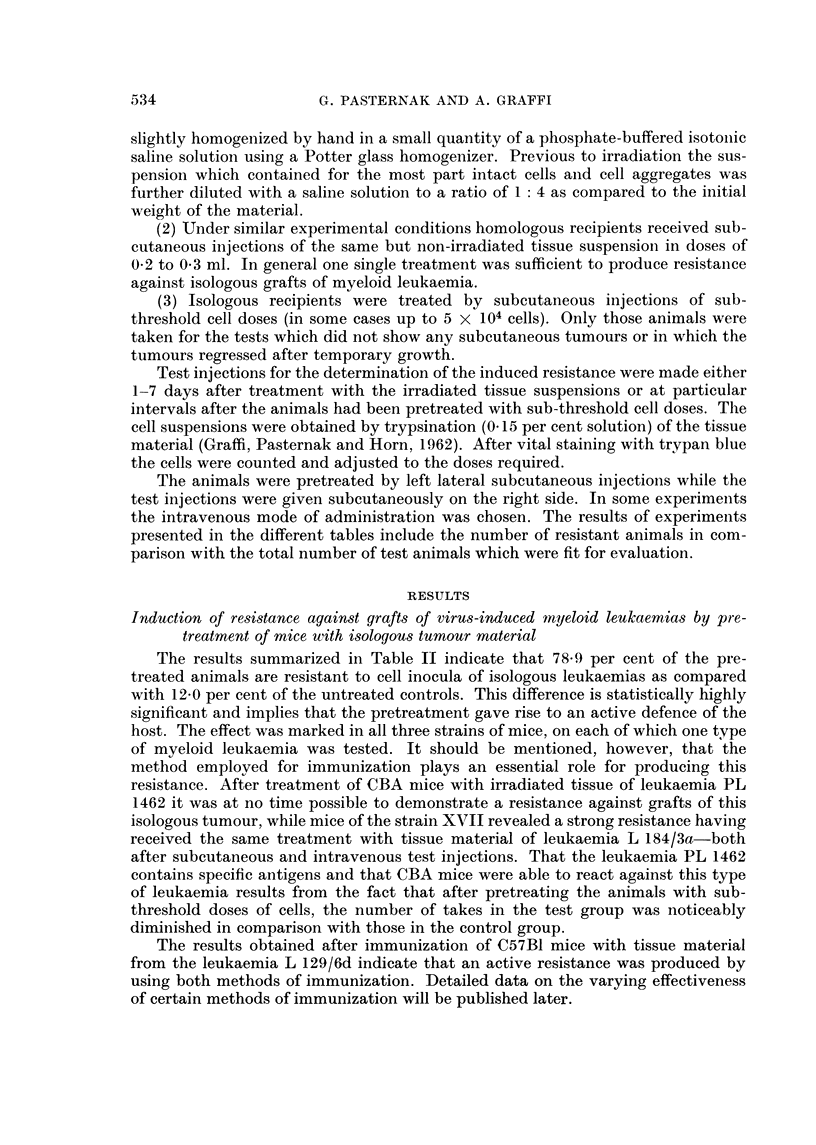

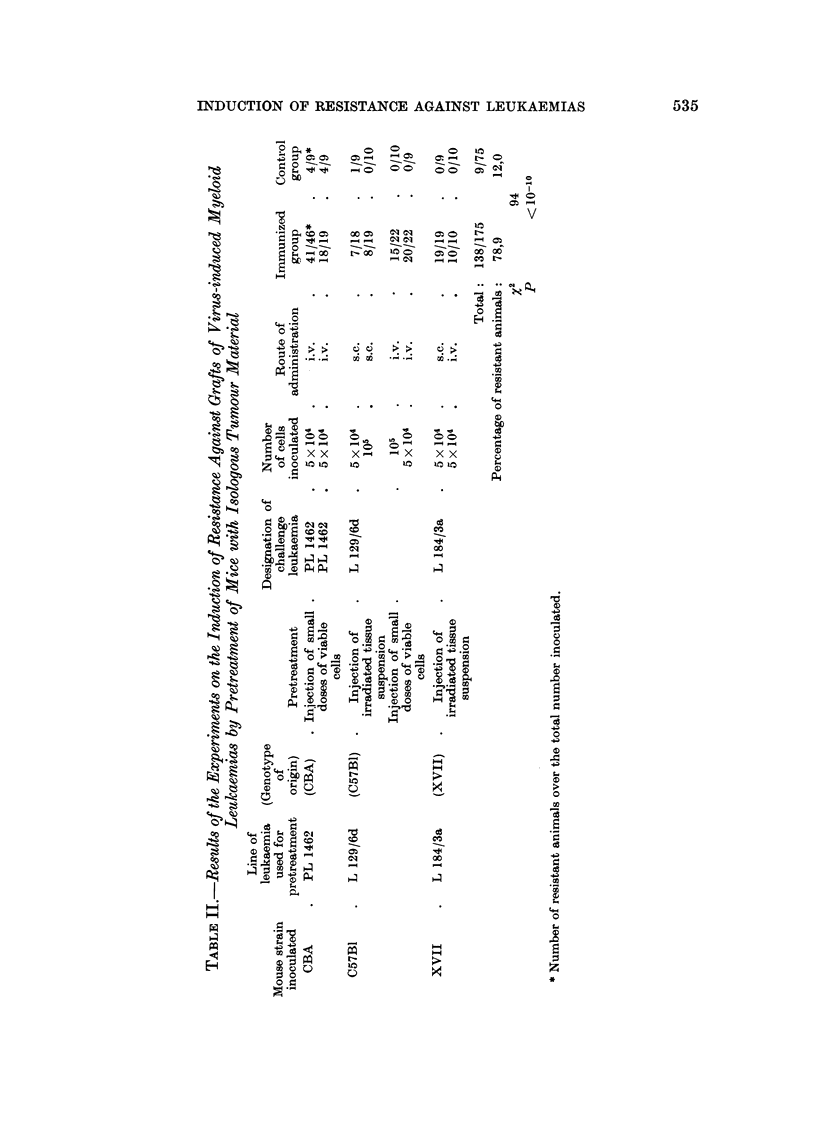

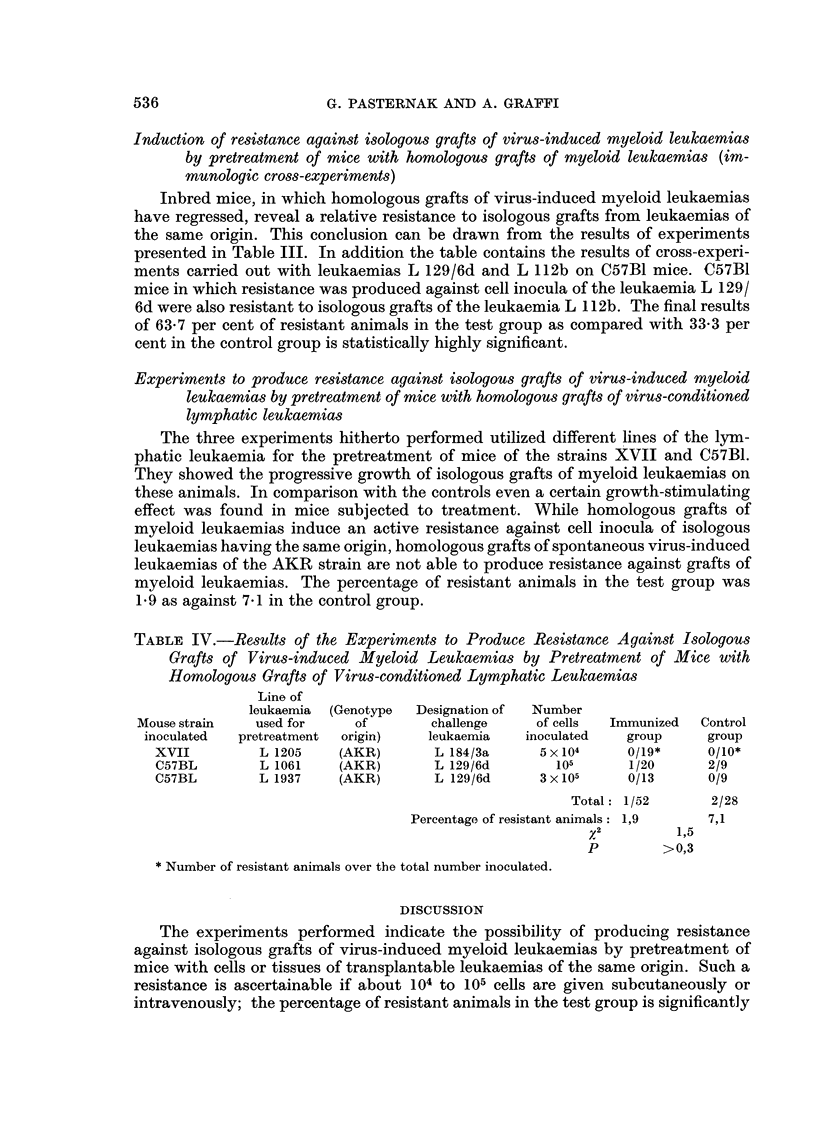

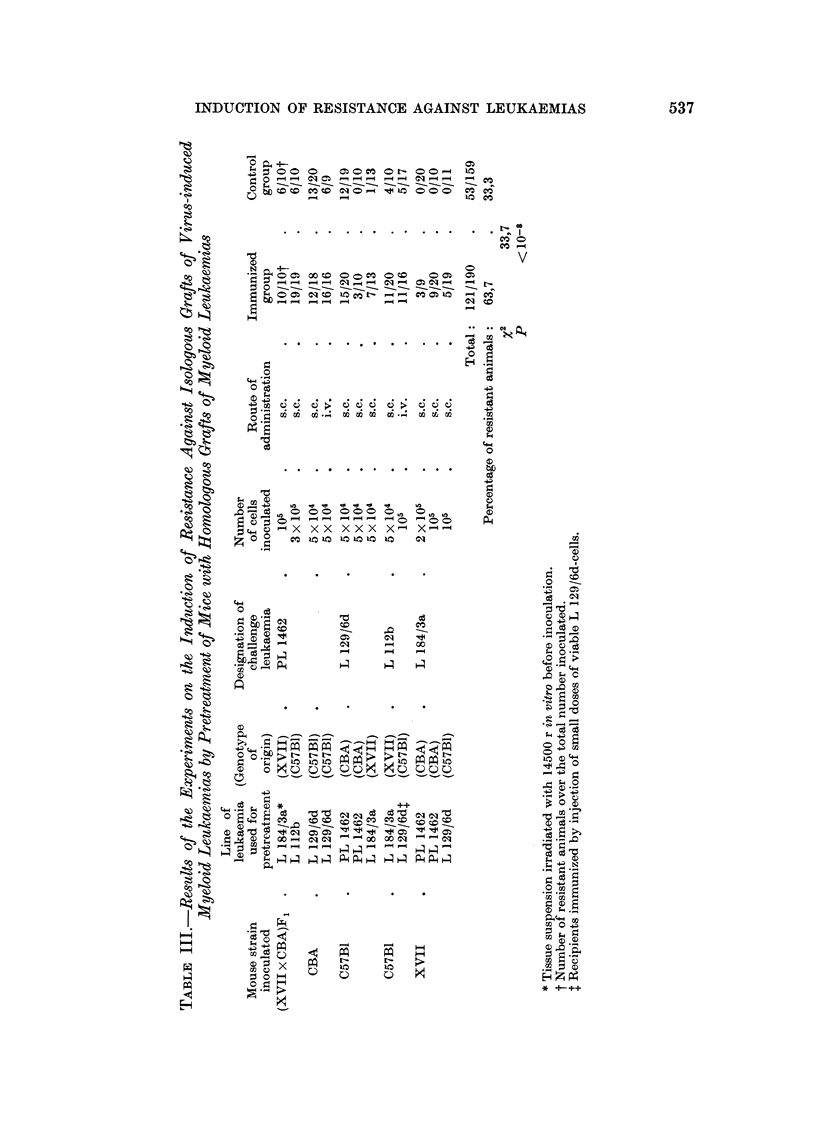

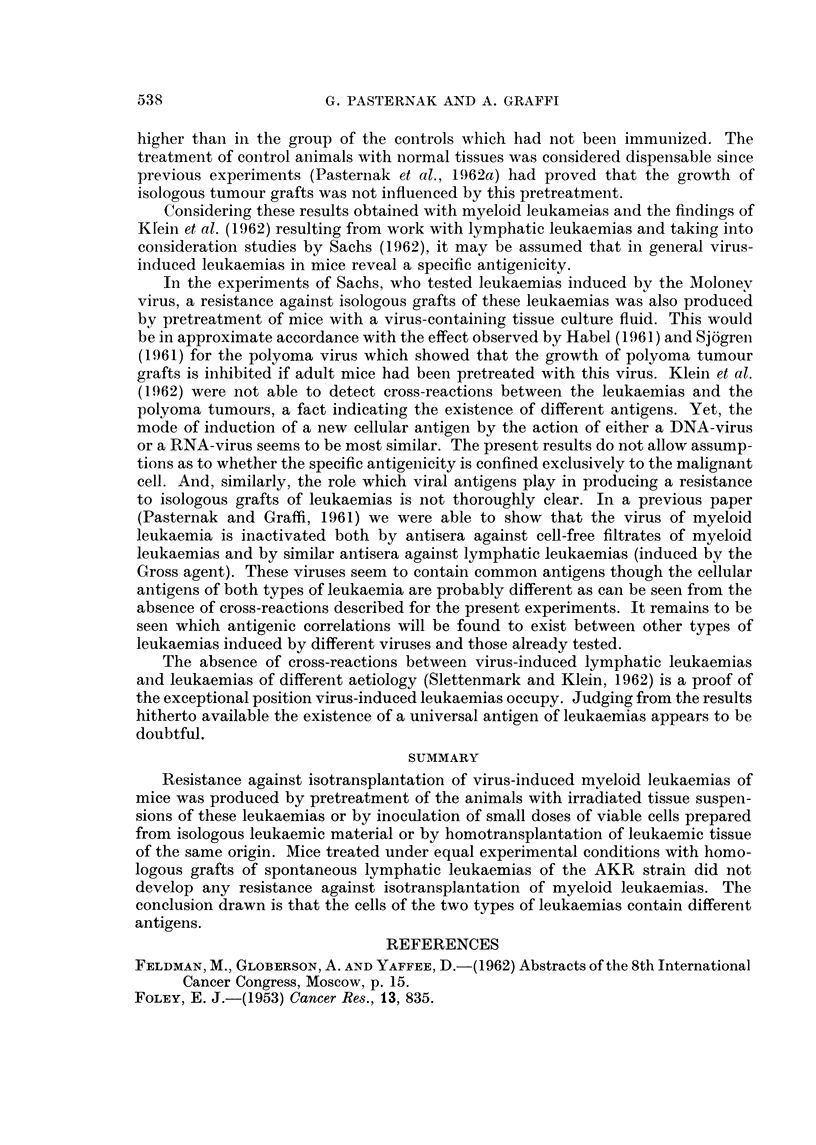

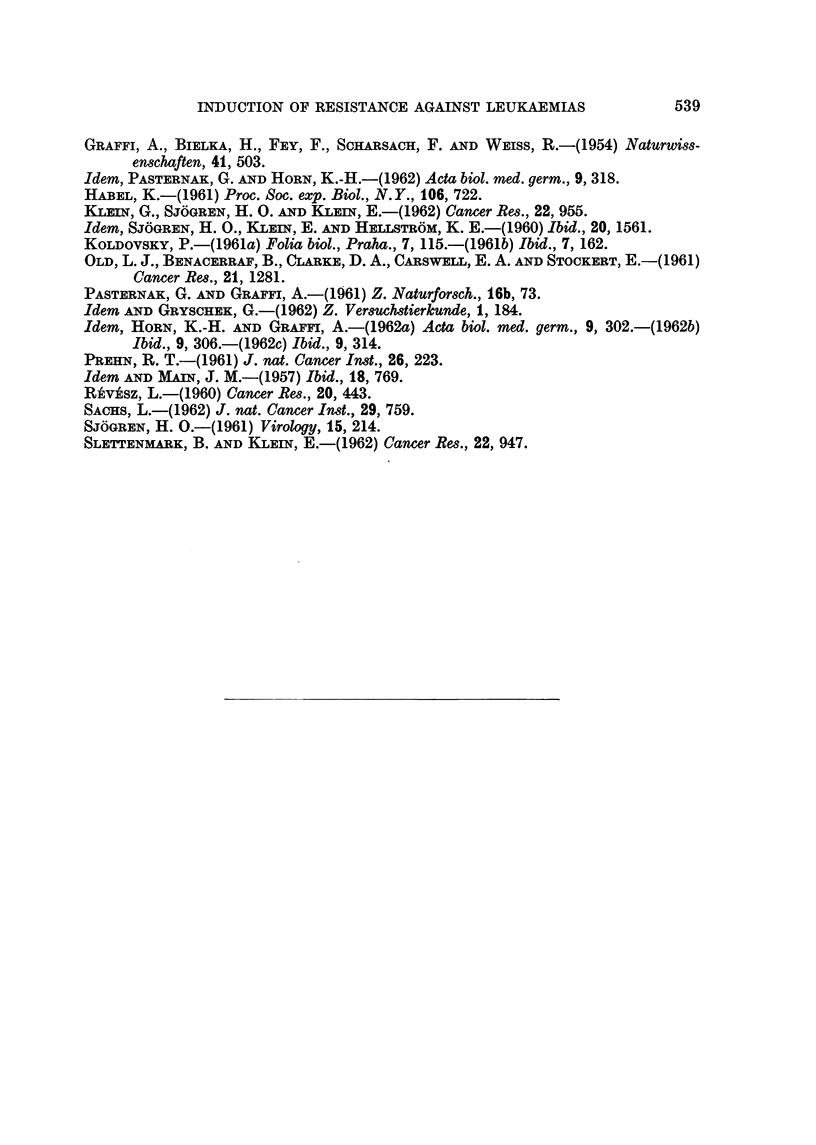

